# The role of *APOE-ɛ4* and beta amyloid in the differential rate of recovery from ECT: a review

**DOI:** 10.1038/tp.2015.39

**Published:** 2015-03-31

**Authors:** T A Sutton, H R Sohrabi, S R Rainey-Smith, S M Bird, M Weinborn, R N Martins

**Affiliations:** 1 School of Psychiatry and Clinical Neurosciences, University of Western Australia, Crawley, WA, Australia; 2 Centre of Excellence for Alzheimer's Disease Research and Care, School of Medical Sciences, Edith Cowan University, Joondalup, WA, Australia; 3 Sir James McCusker Alzheimer's Disease Research Unit, Hollywood Private Centre, Nedlands, WA, Australia; 4 School of Psychology, University of Western Australia, Hollywood Private Hospital, Nedlands, WA, Australia

## Abstract

Individual biological differences may contribute to the variability of outcomes, including cognitive effects, observed following electroconvulsive treatment (ECT). A narrative review of the research literature on carriage of the apolipoprotein E ɛ4 allele (*APOE-ɛ4*) and the protein biomarker beta amyloid (Aβ) with ECT cognitive outcome was undertaken. ECT induces repeated brain seizures and there is debate as to whether this causes brain injury and long-term cognitive disruption. The majority of ECT is administered to the elderly (over age 65 years) with drug-resistant depression. Depression in the elderly may be a symptom of the prodromal stage of Alzheimer's disease (AD). Carriage of the *APOE-ɛ4* allele and raised cerebral Aβ are consistently implicated in AD, but inconsistently implicated in brain injury (and related syndromes) recovery rates. A paucity of brain-related recovery, genetic and biomarker research in ECT responses in the elderly was found: three studies have examined the effect of *APOE-ɛ4* allele carriage on cognition in the depressed elderly receiving ECT, and two have examined Aβ changes after ECT, with contradictory findings. Cognitive changes in all studies of ECT effects were measured by a variety of psychological tests, making comparisons of such changes between studies problematic. Further, psychological test data-validity measures were not routinely administered, counter to current testing recommendations. The methodological issues of the currently available literature as well as the need for well-designed, hypothesis driven, longitudinal studies are discussed.

## Overview

Electroconvulsive treatment (ECT) of mental illness is a ‘Blind Tool': when, how, why and if it works, is unpredictable. The diseases, disorders, syndromes and conditions treated by electrical-induced convulsions have few commonalities, or measured aetiologies; the mechanisms of its actions upon the brain are known imprecisely, and its causal relationship to outcome is unknown. Yet, ECT is demonstrably effective in the relief of some of the major psychopathologies.

This review focuses on genetic, protein biomarker and neuropsychological research associated with the variable ECT recovery patterns observed in the literature. Specifically, the potential utility of conceptualising ECT as controlled, transient minimal brain change is considered. In addition, evidence for the relationship between the variable ECT recovery patterns and individual biological differences as determined by carriage of the apolipoprotein E ɛ4 (*APOE-ɛ4*) allele and by levels of the protein biomarker beta amyloid (Aβ) will be discussed. This review aims to identify the next steps for future research and potential additions/modifications to protocols for ECT use in clinical environments, as well as possible complications and health benefits in patients over the age of 65 years treated with ECT.

## Brief biography of ECT

Convulsions caused by electrical stimulation were first used in 1938 on an individual with schizophrenia suffering delusions and hallucinations, by Cerletti and Bini.^1^ The patient was reported as having received a beneficial seizure, which restored him to ‘clear-headedness' and health. According to Aruta,^2^ however, in contrast with the published account of the patient simply having a ‘successful' seizure, he had three failed prior attempts at inducing a seizure, each with increasing voltages. Further, it is reported that the patient “shouted at Cerletti not to administer the ‘deadly' shake…”, and while Cerletti and Bini described the patient as making a full recovery, he actually relapsed 2 years later. Hence, variable recovery following ECT has been noted from the start.

In the 1930s, Meduna, and others, classified what we now term mood disorders with the depressive and anxious expressions of those with the alternating stupor and excitement of catatonic schizophrenia.^3^ Gradually, ECT was seen to especially benefit depression,^1, 4^ although depression may or may not have been a discrete disorder. Lothar Kalinowsky (1899–1992), a German psychiatrist, during the same early period, urged that ECT should not be used on those with character disorders and anxiety, again defined by the theories of the moment.

ECT is usually now used only when other treatments have been ineffective, poorly tolerated or there is a need for rapid change, such as acute and intense suicidal behaviour. ECT is recommended for use by the American Psychiatric Association Task Force on ECT,^5^ Royal College of Psychiatrists Special Committee on ECT^6^ and the Institute for Health and Clinical Excellence^7^ for the following diverse conditions:

- Severe depression (unipolar and bipolar)
- Acute mania (and bipolar mixed states)
- Schizophrenia
- Catatonia

Further, ECT has been approved by the US' Food and Drug Administration for use in depression, unipolar and bipolar disorders, schizophrenia, bipolar manic and mixed states, schizoaffective disorder, schizophreniform disorder and catatonia.^8^

The original ECT ‘…devices used 125 volts, 50 hertz line current…modified only by a simple mechanical timing mechanism based on a metronome'.^9^ The current procedures used for the administration of ECT have not changed in principle since Holmberg and Thesleff published their protocol in 1952 advocating the use of succinyl-choline-iodide to produce muscular relaxation and reduce the convulsions without the risk of prolonged respiratory difficulties.^10^ (Of note, succinylcholine mimics the choline end of acetylcholine, making it an acetylcholinesterase inhibitor.^11^ The latter is targeted by donepezil, rivastigmine and galantamine, drugs commonly used in the treatment of early stages of Alzheimer's disease (AD). The hypothesis is that lower concentrations of acetylcholine are found in those with AD; hence, keeping levels up might be useful. If a depressed ECT patient is in prodromal AD, the use of any acetylcholinesterase inhibitor becomes a confounding factor in teasing out the efficacy of variables.) The intervening years have seen three changes in the technique: the use of anaesthesia and muscle relaxants, brief and ultra-brief pulse stimuli and unilateral non-dominant hemisphere electrode placement options. These replaced the bite guard, raw current and bilateral placements. Entry and exit points of electric current are either bi-frontal or unilateral, usually on the right side. In any event, regardless of electrode placement, wave shape and pulse interval, a generalised seizure is generated producing widespread bursts of abnormal excitatory synaptic activity in the central nervous system.

## ECT, brain injury and cognitive change

There is debate surrounding the issue of whether the transitory neural changes caused by ECT-induced seizures produce long-term structural or neurochemical alterations contributing to cognitive impairment. The studies reviewed below highlight variable results with respect to both the questions of whether ECT causes any form of brain injury; and whether ECT causes any form of permanent cognitive change. The last issue has two parts: whether cognitive changes, if they occur, are positive or negative. For example, Kroes *et al.*^12^ showed that one dose of ECT prevents the adequate consolidation of reactivated negatively charged episodic memory after a time delay of 1 day (raising the possibility of modifying negatively emotionally charged episodic memories), whereas a raft of other studies show gross autobiographical memory loss as an undesired outcome.^13^ Methodological variations, which will be discussed in detail later, may contribute to the varying results reported.^14^

## Seizure-induced brain injury

Sommer, collecting others' autopsy reports and his own observations, found that in 90 cases between 1825 and 1880, of individuals with a history of seizures due to epilepsy, 30% had pathological changes in the hippocampus.^15^ Sommer's sector refers to a region of Ammon's horn within the hippocampus where he described in detail severe neuronal loss. The hippocampus is associated with memory formation and its dysfunction is evident in early AD.

Inherent features of the hippocampal neuron are proposed to account for seizure-induced cell death.^15^ The neurons of Sommer's sector (CA1) and CA3/4 have more receptors for the excitatory neurotransmitter glutamate than neurons in the more resistant sectors of the hippocampus.^16^ An overabundance of glutamate released at synaptic terminals during disease states such as epilepsy can be toxic due to a sudden and detrimental postsynaptic Ca^++^ influx, and neurons especially prone to cell death are richer in glutamate receptors (causing the so-called glutamate excitotoxicity). Aggravating the tendency for neurons to succumb to excitotoxicity, the more vulnerable neurons of CA1 and CA3/4 have less Ca^++^ buffering capability, due to small concentrations or absence of calbindin and chromogranin A compared with the more resistant neurons of CA2 and granule cells of the dentate gyrus.^17^ Therefore, a poor ability to protect against the excitotoxic effects of glutamate subsequently leading to cell death, by Ca^++^ influx, is the pathogenic mechanism of Ammon's horn sclerosis.^15^

Seizures induce many changes in gene expression in the nervous system, which can lead to the development of chronic epilepsy and/or neurodegeneration.^18^ It is generally acknowledged that convulsive status epilepticus leads to brain injury.^19, 20, 21, 22^ Further, there is growing evidence that non-convulsive status epilepticus can also lead to brain injury.^23, 24^

Work on animal models indicates that brief single seizures can lead to apoptotic neuronal death bilaterally in the rat dentate gyrus.^19^ Complex and simple partial seizures without convulsions cause increased risk for new or increased neurological deficit.^23^ Further, it has been shown that, even without attendant hypoxaemia, acidosis, hyperthermia and hypoglycaemia, ongoing seizures in primates and rats can cause neuronal death.^25, 26^

A review of human research involving neuropathological studies, magnetic resonance imaging and magnetic resonance spectroscopy of epileptic patients experiencing repeated brief seizures indicates brain injury occurs more often than not.^20^ Hippocampal sclerosis is evident in two-thirds or more of hippocampal specimens from temporal lobe epilepsy (TLE) patients (*n*=572), those with extra-temporal seizures and pathologies (*n*=73) as well as among anterior temporal lobectomy and hippocampectomy specimens from patients with medically intractable TLE (*n*=183).^27, 28^ Hippocampal volume loss is reported after seizures,^29^ whereas TLE, the most common of the transient seizure syndromes, results in widespread atrophy including the hippocampus, occipitotemporal areas, cerebellum, cingulate cortex, the ipsilateral insula and thalamus.^30^ Sutula *et al.*^20^ summarises their review of the literature by stating that ‘The emerging perspective is that seizure-induced damage should be regarded not only as neuronal loss but as adverse long-term behavioural and cognitive consequences'. Whether such pathology manifests following ECT-induced seizures, however, remains to be determined.

## Studies of ECT and neural injury

ECT studies have produced apparently more optimistic findings as to whether injury to the brain occurs following the type of repeated seizures ECT procedures induce. A number of studies found no neuronal injury following bilateral ECT,^31, 32^ whereas others report that ECT can have a ‘positive effect on restoring grey matter volume' in treatment-resistive depression.^33^ Restoration to normal levels of cortical glutamate/glutamine^34^ has been described, whereas others conclude that there ‘is no credible evidence that ECT causes structural brain damage'.^35, 36^ Interestingly, a case report detailing post-mortem brain examination of an 84-year-old man who had undergone 422 ECT sessions described no significant microscopic abnormalities, once again suggesting that ECT is not associated with identifiable structural brain injury.^37^ Despite this apparent consensus regarding a lack of association between ECT and neural injury, methodological variations exist between the studies described above suggesting that caution is required when interpreting such findings; these variations will be discussed in a later section.

## Cognitive change

In contrast to the general agreement between reports of ECT and absence of neural injury, studies of cognition have produced conflicting results as to the presence or absence of prolonged cognitive change following ECT. It has been reported that individuals referred for ECT due to drug-resistant depression, when matched and compared with those with depression but not referred for ECT, produced different cognitive profiles.^38^ The former were weaker on executive tasks, the latter on memory, suggesting subtypes of depressives with dissimilar baseline cognitive profiles to begin with. Numbers were small (*n*=15 in each group) and all were female. An exceptionally detailed neuropsychological evaluation of the long-term outcome of ECT and cognitive change in those with bipolar disorder found significant verbal memory impairments at 6 months post bilateral ECT.^39^

In a review of 84 studies, Semkovska and McLoughlin^40^ concluded that cognitive disruption occurs over 3 days post ECT and subsequently resolves, and that after 15 days there is improvement in speed of processing information, working memory, anterograde memory and some executive functions above baseline.

A seminal study found electrode placement position to be important in causing more or less cognitive disruption at 6 months follow-up, with bilateral placement the more disruptive.^13^ Older age was correlated with poorer outcome, and greater cognitive reserve with better outcome. Lack of control groups prevented any conclusions about post-ECT cognitive recovery returning to premorbid levels. This study is rare in its use of reaction time as a measure of speed of processing. The authors also found longer waveforms to be more deleterious in terms of cognitive disruption.^13^

Following on from the earlier work of Sackheim *et al.*,^13^ Verwijk *et al.*^41^ conducted a review of 10 studies focusing on cognitive outcomes following brief and ultra-brief pulse ECT. The authors concluded that autobiographical memory and word fluency (word finding) were impaired immediately post ECT, and to a lesser extent up to 6 months post ECT. Conversely, verbal memory, visual scanning and symbol-coding performance returned to baseline or improved. The authors noted, however, that there were too few studies to draw conclusions about cognitive functions other than memory. Overall, Verwijk *et al.*^41^ could not fully conclude that cognitive functions returned to premorbid levels at 6 months post ECT treatment.

Loo *et al.* concluded that although ultra-brief ECT likely causes less cognitive impairment than other pulse width regimes, some cognitive impairment may still occur. The authors also noted that treatment efficacy is not yet demonstrated and therefore this approach should only be used in research designs.^42^ Sienaert *et al.*,^43^ by contrast, using an extensive range of neuropsychological tests, concluded that no dysfunction occurs following ultra-brief ECT. Unfortunately, however, the same tests were given on three occasions with no allowance for practice effects or use of a reliable change index correction.

The major complaint of some patients following ECT is the loss of autobiographical memory. This is a failure of the episodic memory system^44^ as distinct from semantic memory. The former refers to recalling an event in the past that one is aware to have occurred in the past to oneself. Retrieval from the semantic system is the recall of factual material, and does not require awareness of one's own experience of that fact in the past. For example, recalling what was eaten for breakfast yesterday is episodic, remembering the pulse time distinction between brief and ultra-brief ECT is semantic. The dissociation between episodic and semantic systems is seen in case studies of patients with traumatic brain injury.^45^

Fraser *et al.*^46^ reviewed 15 studies of ECT and autobiographical memory and came to mixed conclusions due to the difficulty in establishing the construct validity of the various subjective autobiographical memory measures used in the studies. The authors suggest that levels of autobiographical memory loss correlate with the type of treatment procedure: bilateral and sine wave ECT administration had a more deleterious effect than unilateral and brief pulse procedures. As noted above, Verwijk *et al.*^41^ did find that autobiographical memory was impaired immediately post ECT, and to a lesser extent, but still present, up to 6 months post ECT.

Memory loss following ECT might be desirable. Treatment of psychological disorders often involves manipulating episodic memories (cognitions, thoughts and beliefs) to some degree and in some fashion. Theoretically, loss of distressing memories will cause loss of the associated negative effect. Kroes *et al.*, while investigating the recall process of negatively charged episodic memory, found the consolidation of an earlier learned distressing event (stories and visuals) was disrupted following a single session of ECT if the memory was ‘reactivated' just prior to ECT and measured 1 day post ECT.^47^ Causation of the disruption to memory consolidation in this study could not be factored out, as either the seizure and/or anaesthetic could be involved.

A more recent retrospective case-note study (*n*=126), however, indicated that patients benefited from ECT on global cognition (as measured by the Mini Mental State Examination) but experienced transient cognitive decline within the first 3 months, as indicated in the spatial recognition memory subtest of the Cambridge Neuropsychological Test Automated Battery.^48^ Possible explanations of the conflicting results reported regarding the presence or absence of prolonged cognitive change following ECT will be discussed in detail in a subsequent section.

## Depression, the elderly and dementia

Depression, mild cognitive impairment (MCI) and AD are linked, with MCI seen as a potential prodromal stage of AD.^49^ MCI is a syndrome where cognitive decline is greater than expected for the person's age and educational level but activities of daily living are not significantly impacted.^50^ Statistically, a portion of the elderly with depression receiving ECT may develop either MCI or AD. Depression occurs in 8–16% of the older community.^51, 52^ The elderly, defined as either over 60 or 65 years of age, comprise a significant group receiving ECT, and are over-represented compared with younger age groups.^14, 53^

Depression as a risk factor for AD was seen in a retrospective study.^54^ Despite the study being based on the recall of subjects, more diagnoses of depression were found in those who developed dementia than in controls, on average some 6 years prior. This is a consistent finding in all subsequent research. Diniz *et al.*,^55^ in a systematic review and meta-analysis of 23 studies found late-life depression was associated with an increased risk for various dementia syndromes including vascular dementia and AD.

Hausner *et al.*^56^ found ECT to be safe for older (65–89 years) depressed patients, regardless of a diagnosis of dementia, MCI or no cognitive impairment. Hausner *et al.* concluded that ECT does not induce cognitive deficits, regardless of baseline cognitive status. The latter was measured by the Mini Mental State Examination, a global screening measure of cognitive status with low ceiling effects and relatively poor sensitivity to subtle cognitive changes, 6 months post ECT. The test was repeated three times at the sixth-treatment, 6-week and 6-month marks, making no allowance for practice effects. Samples were small for the cognitively impaired groups: MCI (*n*=19) and dementia (*n*=12) with the range of scores at 6 months post ECT between 20–30 for the MCI group, and 22–29 for the dementia sample. This indicates wide variability in functioning among cognitively impaired groups, and some individuals with dementia were not scoring as such on the test.

Other studies of ECT in the elderly also suffer from design limitations. For example, while attempting to compare bilateral with unilateral electrode placement, one study was able to reassess only 35% of the subject group due to refusal or lack of consent capacity, ensuring that cognitive changes in the majority of the sample could not be evaluated.^57^ A Cochrane review aimed at assessing the efficacy and safety of ECT in depressed elderly could find only four randomised studies meeting their criteria, all of which had serious methodological problems, preventing any conclusion being drawn. It was therefore not possible for the authors to determine whether ECT is more effective than antidepressants, nor could any conclusion be made as to the safety or side effects of ECT in the depressed elderly.^58^

Moreover, depression is conceptualised and measured in each study quite differently. Simple screening measures or symptom checklists specific to depression are routinely utilised. In MCI, depressive symptoms may also be those of cognitive change; for example, sleep disturbance, poor concentration and anhedonia:^59^ these factors make it difficult to conceptually disentangle a depressive from a cognitive symptom. Uniform utilisation of more comprehensive assessments may facilitate differential diagnosis, and further work in the area of ECT, depression, the elderly and dementia is required.

## ECT and *APOE-ɛ4*

Apolipoproteins such as ApoE bind to low-density lipoprotein receptors thereby mediating transport of cholesterol and other lipoproteins. This transported cholesterol is used to make dehydroepiandrosterone, sex hormones and the glucocorticosteroids. The latter, at elevated levels, mimics the effects seen in post-traumatic stress disorder in the hippocampus, and negatively affects memory.^60, 61^ ApoE is encoded by the *APOE* gene, located on chromosome 19, which has three major alleles: ɛ2, ɛ3 and ɛ4, related to amino-acid substitutions (Arg and Cys) at positions 112 and 158 of the protein. These single amino-acid substitutions result in significant functional differences. The frequency of *APOE* alleles in the Australian population is similar to other western countries: *APOE-ɛ2* 8%, *APOE-ɛ3* 78% and *APOE-ɛ4* 14%.^62^

Possession of the *APOE-ɛ4* allele has become firmly established as a risk factor for the incidence of late-onset AD (LOAD).^63^ The frequency of the ɛ4 allele in familial LOAD was found to be 50% compared with 14–16% for controls^64^ and 40% in those with autopsy-confirmed AD pathology but no family history.^65^ AD risk and *APOE-ɛ4* carriage is dose dependent: individuals who are heterozygous and homozygous for *APOE-ɛ4*, have a threefold and eightfold increased risk of developing LOAD by age 80 years, respectively. Importantly, however, 50% of those who are homozygotic for *APOE-ɛ4* survive up to the age 80 years without cognitive impairment, such that the possession of this genetic configuration is not deterministic. Levels of cerebral Aβ deposition, a hallmark of AD, are directly related to inheritance of an ɛ4 allele, although the increased Aβ deposition observed in ɛ4/ɛ4 and ɛ3/ɛ4 patients is not related to survival times.^63^

The *APOE* gene while primarily expressed in hepatic parenchymal cells,^66^ is also expressed in the brain, where the ApoE protein is produced mainly by astrocytes. Astrocytes synthesise ApoE, which combines with cholesterol and phospholipids to form lipid–protein complexes, that are postulated to maintain and repair nerve cell membranes, aid neuritic growth and facilitate synaptogenesis. The repair process occurs when the lipid–protein complex is released into the extracellular space and taken up by ApoE receptors on the nerve cell surface, before being internalised into the cell. It is thought that ApoE itself transports lipids within the brain, enhances the structural integrity of microtubules within the neuron and possibly facilitates neural transmission.

It has been reported that individuals carrying the *APOE-ɛ4* allele are prone to experiencing a slower recovery from brain injury.^67^ If ECT causes neural injury, then recovery from experiencing ECT should also be prolonged in those who carry the *APOE-ɛ4* allele. Only three studies have examined ECT response and carriage of the *APOE-ɛ4* allele. Interestingly, Fisman^68^ found that those with *APOE-ɛ4* and late-onset depression, but not psychosis, had a better response to ECT than ɛ4 non-carriers, whereas Huuhka *et al.*^69^ found no differences in response to ECT, regardless of *APOE* genotype. In agreement with the results of Huuhka *et al.*, Bousman *et al.*^70^ also reported no association between *APOE* genotype, treatment efficacy and cognitive side effects of ECT among 117 participants with major depressive disorders undertaking ECT through clinical study participation. Bousman *et al.*^70^ concluded that the younger age of their cohort may account for the disparate results, with *APOE* contingent effects apparent only in older age. Further work is required, however, to validate these findings.

Fisman *et al.*^68^ point out that the good response to ECT in those with *APOE-ɛ4* is ‘counter-intuitive', given the previously cited links between *APOE-ɛ4* Aβ deposition after brain injury,^71^ and its association with AD. To account for the observed response, Fisman *et al.* hypothesise that the *APOE-ɛ4* genotype regenerates proximal dendrites, producing a greater antidepressant effect. The authors base their hypothesis partly upon the work of Arendt,^72^ and partly on the discussions and hypotheses of Duman and Vaidya.^73^ The former, in the view of Fisman *et al.*, showed that those with ɛ3/3 alleles regenerated more distal dendritic segments, whereas ɛ3/4 carriers regenerated both distal and proximal dendritic parts, and ɛ4/4 carriers localised regrowth closer to the soma.^72^ Duman and Vaidya^73^ discuss research on ECT causing an increase in the brain-derived neurotrophic factor, which they hypothesise then reverses the atrophy of stress-vulnerable neurons or protects these neurons from further injury.

Arendt^72^ worked on autopsied brain specimens and observed impairment of neuronal repair in those possessing the *APOE-ɛ4* allele, whereas Duman and Vaidya^73^ reviewed diverse findings from animal and human research. Notably, despite the hypothesis by Fisman *et al.* being speculative, and the drawbacks in their research design, this single study is cited in 10 articles as confirmatory of the link between *APOE-ɛ4* and a successful response to ECT.

The study by Huuhka *et al.*^69^ is methodologically firmer than that of Fisman *et al.*^68^ All patients received the same ECT procedure (bilateral) and the outcome criteria was defined by clearly separating good and poor responders with non-lapping scores on the Montgomery–Asberg depression rating scale: <8 for responders and >15 for non-responders. As with Fisman *et al.*, there were mixed diagnostic groups: patients with depression with or without psychosis. The patient age range was 22–84 years. Early onset of depression was defined as <45 years and late onset as >45 years of age, and psychotropic medication was present.

In summary, the only three studies in this area are conflictual and suffer multiple methodological limitations. They reflect the difficulty in undertaking ECT research while simultaneously attempting to treat patients within the clinical context.

## ECT and Aβ

Histopathologically, LOAD is characterised by senile plaques and neurofibrillary tangles. The latter are aggregates of the hyperphosphorylated tau protein. The plaques are mainly composed of aggregated Aβ peptides derived from the amyloid precursor protein encoded on chromosome 21. AD is therefore, likely to be at least in part, a function of beta-protein amyloidosis. Aβ is suggested by some to be important in maintaining synaptic function, learning and memory,^74^ with its causal role in AD overemphasised.

Aβ, first described in 1984,^75^ results from proteolytic cleavage of the amyloid precursor protein, of which two short amino-acid sequences, Aβ_1__–40_ and Aβ_1__–42_ are products.^74^ Aβ_1__–42_ is more amyloidogenic than Aβ_1__–40_.^76^ Although Aβ_1__–42_ amino-acid production is, overall, less common than the 1-40 type, it is the dominant kind of Aβ found in the amyloid plaques of those with AD.

Only two studies of Aβ levels following ECT could be found. Zimmerman *et al.*^77^ measured plasma Aβ peptides 1-40 and 1-42 before ECT, ~30 min post ECT and at ~2 and 24 h after ECT treatment in 13 patients. The average age of the sample was 50.86 (s.d. 14.5 years). The diagnoses included depressive episodes in bipolar affective disorder or in recurrent depressive disorder with or without psychotic symptoms (seven patients), and depressive type of schizoaffective disorder. The brief-pulse stimulation technique was applied with right unilateral stimulation in each patient.

The authors found a significant increase in the plasma concentrations of each Aβ species ~30 min after ECT, followed by normalisation of the peptide's concentrations 2 h after ECT. Specifically, elevations in Aβ following ECT at 30 min were between 5 and 10% from baseline, normalised at 2 h and remained normal at 24 h. Aβ levels were not correlated with the number of ECT sessions or the electroencephalogram-measured seizure duration.

Further, in a first study to analyse Aβ before and after ECT in 25 drug-resistant bipolar depressed patients, Aβ_1_–_40_ and Aβ_1__–42_, as well as their relative ratio were measured before and after 3–4 weeks of ECT.^78^ Although the authors were not able to identify changes in plasma concentrations of these isoforms post treatment, they did observe a positive correlation between Aβ_1__–40_/Aβ_1__–42_ ratio and severity of depressive and cognitive symptoms, as determined by Clinical Global Impressions-Severity of Illness Scale score and the Hamilton Rating Scale for Depression (HRSD-21) score; that is, the higher the biological parameters, the more severe the condition. Furthermore, individuals who entered clinical remission after ECT presented a significantly lower Aβ_1__–__40_/Aβ_1__–__42_ ratio at baseline, compared with non-remitters, suggesting a possible method of characterising depressed individuals who may respond positively to ECT.

One difficulty in interpreting these results is that, whereas plasma and cerebrospinal fluid (CSF) measures of Aβ_1__–__40_ and Aβ_1__–__42_ might correlate in healthy subjects, they might not in those with AD, where CSF levels are the more discriminatory.^79, 80, 81^ To date, no study has reported Aβ levels in the CSF following ECT. Such a study could have significant scientific impact on our understanding of the potentially damaging or beneficial effects of ECT in relation to a molecule heavily implicated in AD neurodegeneration.

If the depressed elderly benefit from ECT, and a proportion have prodromal AD, and if cognition improves, then a causal mechanism between seizure activity and cognitive improvement should exist. If AD is correlated with increased Aβ, then one hypothesis is that this protein is affected by ECT: there should be less Aβ in the brains of successfully ECT-treated older dementia patients. Positron emission tomography imaging utilising Aβ_-_binding radiolabelled tracers (for example, carbon-11- labelled Pittsburgh Compound-B; C11-PiB) yielding estimates of cerebral Aβ load would be required to test this hypothesis. This relationship has not been previously examined and should, where possible, be included in future studies of ECT and Aβ. Further, amyloid imaging can serve as an outcome measure if proved to be significantly associated with ECT application.

## Methodological variations as potential confounders

Despite the apparent consensus regarding a lack of association between ECT and neural injury, methodological variations between studies quell the robustness of such a conclusion. The studies showing no brain injury results from ECT, suffer problems of patient heterogeneity with variable diagnoses, large age ranges (20–80 years), gender and educational differences. Further limitations are imposed by small sample sizes; variable numbers of ECT sessions; bilateral, unilateral, brief and ultra-brief ECT procedures; and various biological markers assessed after variable times (from the initial treatment session to some days following).

Inter-study methodological variations may also provide a possible explanation for the conflicting results reported regarding the presence or absence of prolonged cognitive change following ECT. Few studies have control groups of any sort, and even fewer control for confounding variables such as the type of anaesthetic used. In addition, cognitive reserve or premorbid estimates of functioning were measured in only two studies;^13, 82^ the depressed elderly are often combined with younger age groups in the data analysis. Further, the elderly are prone to depression^83^ that in turn can be a prodromal stage risk factor for AD, which is itself evidenced by severe cognitive dysfunction.^84, 85^

Cognitive change covers many functions, including various types of memory, speed of information processing, language processing, executive functions and reasoning. Neuropsychological testing requires a validity measure or measures, assessing whether optimal cognitive performance and hence valid data, have been obtained.^86^ Historically, the term ‘symptom validity test' or ‘response bias' and later the 'performance validity test'^87, 88^ was used to describe such measures. However, no such measure was obtained as part of the studies described.

In their review of 84 studies assessing ECT and cognition, Semkovska and McLoughlin^40^ note the problem of researchers collapsing visual and verbal memory data into one, and failing to discriminate between the various memory processes such as encoding, retrieval or learning. The authors identified 22 different neuropsychological tests across the studies reviewed, with four different wordlist tests, two short-story tests, two paired word associate tests and three visual memory tests among the memory measures, highlighting the lack of uniformity among research cognitive measures. Semkovska and McLoughlin^40^ integrated what they themselves categorised as ‘similar tests' in their analysis, such as treating the results from all wordlist tests as one. This approach, however, limits interpretation of results: for example, the Rey Auditory Verbal Learning Test contains discrete and unrelated words, whereas the California Verbal Learning Test has words that can be categorically grouped, changing the potential encoding strategy required. In addition, the authors report that no pre-treatment data were available in any study, although in fact one included a formal estimate of premorbid intellectual functioning for baseline purposes, the National Adult Reading test.^82^ Further, the age ranges in the 84 studies was between 18 and 87 years, contained differing and multiple diagnostic groups of unknown severity, employed different ECT electrode placements and procedures and tested subjects at differing time intervals (between 45 min and 12 months) during and post ECT administration.

The review by Verwijk *et al.* of ten studies focusing on cognitive outcomes following brief and ultra-brief pulse ECT^41^ also incorporated studies using a multiplicity of neuropsychological measures across a range of functional areas (rote visual and verbal memory, visual scanning speed, working memory, executive processing and visuospatial organisation), as well as different inclusion and exclusion criteria for subjects, age ranges between 14 and 91 years, small samples sizes and different points at which post-ECT functions were measured. However, the authors did note that study number and heterogeneity prohibited robust conclusions to be drawn.

The studies of *APOE*, Aβ and ECT also suffer from methodological variations, which limit the interpretability of their findings. For example, the study of *APOE* and ECT by Fisman is marred by a lack of diagnostic patient selection, there being a mix of depression and schizoaffective disorder; differing ECT procedures (unilateral and bilateral) per subject; inter-rater response outcome criteria disagreement in 17.6% of cases; and patients simultaneously being on additional medications affecting brain chemistry. Among Fisman's sample, no successful responder to ECT with *APOE-ɛ4* had psychotic features or early-onset depression. Furthermore, the age range of those with *APOE-ɛ4* when first diagnosed with depression was 54–72 years, and those without the allele, 15–81 years; early and late onset was defined by the median age in these ranges.^68^

Methodological issues, although detrimental, can be properly addressed through detailed, well-designed studies, something long needed in ECT research. Although such methodological variations are not exclusively seen in ECT research, they should be considered when any conclusion is drawn based on the currently available literature.

## Discussion and conclusions

Clinical necessity compels the continued use of ECT, and its use has been increasingly seen in the elderly worldwide. However, ECT's effect on cognitive functions, particularly among older adults, remains decidedly unclear due to the methodological issues in the published research.

First, there is heterogeneity of patients' characteristics, including a multiplicity of diagnoses and variability in their definitions, extreme age ranges, gender ratios, the presence of prodromal or existing neurodegenerative diseases such as AD, variable cognitive reserve capacities among subjects and biological variability such as differences in known genetic risk factors for AD (for example, *APOE-ɛ4*) and/or recovery from acquired brain injury. Second, there is heterogeneity of emotional measures, each usually restricted to one global construct such as ‘depression'. The psychological measures are usually brief screeners, with few items.

Third, there is heterogeneity of cognitive measures. These range from brief, often insensitive global estimates of cognitive functions such as the Mini Mental State Examination, to a variety of word list, story and associative verbal memory tests, a variety of measures of visual memory and visuospatial skills, speed of processing information and executive functions. Only one study utilised reaction-time tasks and one included an estimate of premorbid level of functioning (or possible cognitive reserve). It is rare for practice effects to be addressed; for example, by using parallel forms of tests or utilizing reliable change indexes based upon the psychometric properties of the instrument (error ranges). There is also complete absence of performance validity test measures, which are required to ensure cognitive data are valid.

Finally, there has been heterogeneity of experimental procedures: cognitive and biomarker valuations are conducted at different times during and post ECT, ranging from minutes to months. Control groups, including sham ECT or epileptic subjects, are poorly selected or absent.

The practical difficulty of ECT research stems from the conundrum posed by clinical urgency. ECT is only used, and is only acceptably used, in intractable and severe cases of mental suffering. Research to date has typically targeted experimental control over clinical application—for example, dropping atypical cases to ensure ‘outliers' are not contaminating any effect. Case studies where an experimental *n*=1, are more practical and may yield additional useful information. This approach is also better suited to research a highly specific question (for example, is CSF Aβ level changed following ECT?) with a specific patient (specified age and a measured diagnosis with construct validity) utilising relevant cognitive outcome measures (using theoretical terms compatible with the psychometric measures).

Standardisation of measures of cognitive functions and biomarkers relevant to known changes in an older person (over 65 years), within a comprehensive, single-case repeated measures design, would yield a sound basis for evaluating the cognitive changes, positive, negative or none, between studies and research groups. This would also allow the incorporation of data from individual clinicians.

An agreed standardised evaluation protocol of older patients subjected to ECT would be a useful precursor to investigating the benefit or cost of induced seizure activity on cognition. Clinically, this may then serve to better identify patients suitable or unsuitable for the procedure.

Finally, although such methodological variability between studies suggests that caution should be employed when interpreting the results, the difficulties associated with conducting such research must not be overlooked. Having said that, there is a long-lasting need for properly designed clinical studies with well-defined samples to elaborate on both cognitive and biological markers potentially associated with ECT. Such a study can address cost/benefit problems while providing information on duration, intensity and dose-dependent effects/side effects of ECT as a therapeutic intervention.

## Notes

RNM is the founder and owns stocks of Alzhyme. HRS has previously received payments from Wyeth and Pfizer, and is currently receiving remuneration for ongoing work as part of the Takeda Pharmaceutical TOMMORROW Study (TOMM40 Trial). The remaining authors declare no conflict of interest.
